# Photodynamic effect of TPP encapsulated in polystyrene nanoparticles toward multi-resistant pathogenic bacterial strains: AFM evaluation

**DOI:** 10.1038/s41598-021-85828-9

**Published:** 2021-03-24

**Authors:** Zuzana Malá, Ludmila Žárská, Lukáš Malina, Kateřina Langová, Renata Večeřová, Milan Kolář, Petr Henke, Jiří Mosinger, Hana Kolářová

**Affiliations:** 1 Department of Medical Biophysics, Faculty of Medicine and Dentistry, Palacky University in Olomouc, 775 15 Olomouc, Czech Republic; 2 Department of Microbiology, Faculty of Medicine and Dentistry, Palacky University in Olomouc, Olomouc, Czech Republic; 3 Department of Inorganic Chemistry, Faculty of Science, Charles University, Prague, Czech Republic; 4 Department of Medical Biophysics, Institute of Molecular and Translational Medicine, Faculty of Medicine and Dentistry, Palacky University in Olomouc, Olomouc, Czech Republic

**Keywords:** Biophysics, Molecular biophysics, Nanoscale biophysics, Single-molecule biophysics

## Abstract

Photodynamic inactivation (PDI) is a promising approach for the efficient killing of pathogenic microbes. In this study, the photodynamic effect of sulfonated polystyrene nanoparticles with encapsulated hydrophobic 5,10,15,20-tetraphenylporphyrin (TPP-NP) photosensitizers on Gram-positive (including multi-resistant) and Gram-negative bacterial strains was investigated. The cell viability was determined by the colony forming unit method. The results showed no dark cytotoxicity but high phototoxicity within the tested conditions. Gram-positive bacteria were more sensitive to TPP-NPs than Gram-negative bacteria. Atomic force microscopy was used to detect changes in the morphological properties of bacteria before and after the PDI treatment.

## Introduction

Emerging problems with antibiotic resistance require the development of novel, effective and low-cost methods to avoid bacterial diseases. In addition to several known physical^1,2^, chemical^3^ and biological methods^4^ to treat bacterial inactivation, the most widespread and commonly used method is antibiotic treatment. Although several new antibiotics have been developed in recent decades, none have improved efficacy against multidrug-resistant bacterial strains^5^. Therefore, it is important to develop alternative and effective therapeutic strategies to inactivate Gram-positive and Gram-negative pathogens^6^.

Photodynamic inactivation (PDI) is one of the promising and effective treatment tools against microbial infections, including those caused by multidrug-resistant strains^7,8^. The mechanism of PDI involves of the photogeneration of highly cytotoxic reactive oxygen species (ROS) with short-lifetimes, particularly singlet oxygen O_2_(^1^Δ_g_), via photosensitized reactions (Fig. S1). Briefly, the triplet state of the photosensitizer is quenched by triplet oxygen, and short-living O_2_(^1^Δ_g_) is formed via energy transfer. A single photosensitizer molecule can produce many O_2_(^1^Δ_g_) before it is destroyed; due to this photocatalytic activity, PDI kills microbes more rapidly and at much lower concentrations than biocides, and clinical management is simply achieved by controlling the visible light dose delivered^9,10^. There are many PDI studies applying several cationic and anionic photosensitizers^11–14^.

The typical disadvantage of the application of free photosensitizers not bound to any matrix is the tendency of photosensitizers to aggregate, which significantly reduces the lifetime of the excited states and, consequently, negatively influences the photogeneration of O_2_(^1^Δ_g_) and the sensitivity of their triplet states to competitive quenchers^15^. The binding of a photosensitizer to a nanocarrier represents a versatile and powerful tool for changing/tuning the properties of encapsulated or attached molecules^6,16^.

Recently, tetraphenylporpyrin (TPP) photosensitizer was encapsulated to stable sulfonated polystyrene nanoparticles (TPP-NPs)^17^. TPP-NPs showed effective generation of O_2_(^1^Δ_g_) and were also used for oxygen sensing. Polystyrene nanoparticles are efficient carriers of photosensitizers due to their small diameter and capability to be transported near to the biological targets, which is important due to short diffusion length of O_2_(^1^Δ_g_) .

O_2_(^1^Δ_g_) is released from TPP-NPs shell into the outer environment with longer lifetime (τ_Δ_ ~ 20 μs but shorter diffusion length lr ~ 58 nm in TPP-NPs matrix) compare to O_2_(^1^Δ_g_) in the water (τ_Δ_ ~ 3.5 μs and diffusion length l_r_ ~ 205 nm). The fraction of effectively released O_2_(^1^Δ_g_) is higher for smaller sizes of TPP-NPs (7—162 nm). Accordingly, photooxidation of chemical substrates confirmed higher photoxidation ability for TPP-NPs with smaller sizes of polymer carrier^18^.

Nanoparticles with polystyrene shell protect the hydrophobic photosensitizer against external quenchers and aggregation. The polystyrene shell has high oxygen permeability (2.8 × 10^–7^ cm^2^ s^−1^) and thus allowing triplet states to be quenched exclusively by oxygen.

Several studies have shown that photosensitizers have different photo-antibacterial efficacies on Gram-positive and Gram-negative bacteria^19,20^. The structure of the cell surface of these bacteria plays a critical role in the interaction between photosensitizing agents and bacterial strains. Gram-positive bacterial strains have relatively strong cell walls (15–80 nm) composed of peptidoglycan, the layers of which penetrate the surfaces of linear chains of teichoic acid^21,22^. In Gram-negative bacteria, the cell wall is composed of two or three interconnected layers of peptidoglycan surrounded by an outer membrane, which consists of a phospholipid bilayer. The cell wall of Gram-negative bacteria is thinner (10 nm thick) and less compact than that of Gram-positive bacteria but remains strong, tough, and elastic, giving the bacteria their shape and protecting them against extreme environmental conditions^23^.

There are several microscopic approaches for cell imaging. Scanning and transmission electron microscopy (TEM) are the most widespread techniques for explaining the morphological changes in bacteria^24^. However, these techniques are exhausting and require complex sample preparation, which may affect the dimensions of the cellular structures^25^. In addition to these techniques, atomic force microscopy (AFM) has been found to be an attractive method for examining the surface morphology of biological samples due to its high resolution and less complicated sample preparation procedures^26–29^.

The aim of this study was to compare the antibacterial properties of TPP-NPs toward Gram-positive (methicillin-resistant *Staphylococcus aureus* (MRSA) and sensitive reference strain *Enterococcus faecalis* CCM 4224 (ENCF)) and Gram-negative (ESBL-producing *Klebsiella pneumoniae* (ESBL) and *Pseudomonas aeruginosa* CCM 3955 *(*PSEA)) bacterial strains using the colony-forming unit method with the help of AFM for the characterization of the cell surface changes induced by PDI.

## Materials and methods

### TPP encapsulated in NPs (TPP-NPs)

Highly sulfonated polystyrene nanoparticles TPP-NPs (average diameter 15 ± 7 nm) with encapsulated hydrophobic TPP (5,10,15,20-tetraphenylporphyrin, Sigma-Aldrich) photosensitizer were prepared by a top-down nanoprecipitation method as published earlier^17,30^. Briefly, sulfonated electrospun polystyrene membranes were washed with deionized water until a neutral pH was achieved. Subsequently, wet membranes were immersed in THF with TPP for a few seconds with stirring, then deionized water was added. THF was evaporated under vacuum. Larger microparticles were separated from the NP dispersion by centrifugation. Finally, the NP dispersion was dialyzed for three days at room temperature against water to remove traces of sulfuric acid and THF. The tested concentration of TPP-NPs dispersion was 3 mg/ml, with a concentration of encapsulated TPP 10% (w/w) in NPs giving 5 × 10^–4^ mol/l TPP for stock dispersion. The concentration of NPs was calculated using gravimetric analysis (2.2). The stock dispersion of TPP-NPs (∼3 × 10^13^ NPs/ml) was stored in the dark. The size evaluation and photophysical characterization of TPP-NPs was described in details in previous study^17^.

### Gravimetric analysis

Twenty milliliter samples of NPs were dried at 50 °C to a constant weight. The weight was determined using a GR-200 analytical balance (A&D Instruments Ltd., Japan). Stock concentration of NPs was calculated from mass of dry samples and molar mass (3.2 mg/ml and 6.8 × 10^7^ g mol^−1^) calculated from light scattering experiments published in previous study^17^.

### Dynamic light scattering (DLS)

Particle size and size distributions in water were determined by dynamic light scattering (DLS) on a Zetasizer Nano ZS particle-size analyzer from Malvern. From previous study^17,31^ follows that the presence of encapsulated TPP at all concentrations used had no influence on the morphology or the size of the NPs.

### Used bacterial strains

For our in vitro study, two Gram-positive and two Gram-negative bacterial strains were used. Methicillin-resistant *Staphylococcus aureus* CCM 4591 (MRSA) and the strain *Enterococcus faecalis* CCM 4224 (ENTF) were used as representative Gram-positive bacterial strains, and ESBL-producing *Klebsiella pneumoniae* CCM 2486 (ESBL) and the sensitive reference strain *Pseudomonas aeruginosa* CCM 3955 (PSEA) were used as Gram-negative bacterial strains. The bacteria were inoculated on blood agar (Trios, Czech Republic) and cultivated at 35 °C for 24 h. Fresh colonies of bacteria were used for the experiment. A bacterial suspension was prepared for each antibacterial assay, and the concentration of bacteria in initial stock bacterial suspension was determined measuring the optical density with a densimeter (Densi-La-Meter; LACHEMA, Czech Republic). The final concentration of stock bacterial suspension was in order of 10^3^ CFU/ml.

### Irradiation

For the irradiation, a homemade LED-based light source containing 350 pieces of 5 mm LEDs with emission at 414 nm wavelength was used^32^. The irradiance of the light source was 54 mW/cm^2^. Samples of bacteria with TPP-NPs were exposed to the irradiation for 0.5, 1, 5 and 10 min, which correspond to the specific doses of irradiation of 1.62, 3.24, 16.2 and 32.4 J/cm^2^, respectively. The irradiation doses (E) was calculated using simple equation:

E = P × t; where P is the irradiance (intensity of irradiation) of the light source and t is the time.

The irradiance was measured using an ILT 1700 radiometer of the SED033 sensor (International Light Technologies).

### Photodynamic treatment of bacterial cell suspension

For photodynamic experiments, 10 × diluted stock dispersion of TPP-NPs (∼3 × 10^12^ NPs/ml) were mixed 1:1 with the stock bacterial suspension.

Two milliliters of this dispersion were placed into a petri dish (Ø 35 mm) at room temperature and irradiated for different time with visible light produced by a LED-based light source (414 nm). The bacterial suspensions were irradiated for different time to achieve the total light doses for activation of TPP-NPs. After the irradiation, 100 μl of the dispersion was spread on agar plates. The plates were incubated in darkness at 35 °C for 24 h to allow the individual bacteria to grow and form colonies^30^.

### Sample preparation for the AFM

For the AFM analysis, air-dried bacterial samples were prepared following the protocol described by Robichon et al.^33^. Briefly, 5 µl (10^3^ CFU/ml) of photodynamically treated and untreated cells was spread on a clean glass (Knittel glass) and air dried at ambient room temperature and humidity φ (T = 22 °C, φ = 50%) for 5 min. It has been reported that bacteria remain alive when dried under these conditions^28^. For the AFM imaging, we used the air-dried samples; this mode is generally used for evaluating the change in morphology of bacterial strains caused by antibacterial agents^26,27^.

### AFM imaging

The Atomic Force Microscope Bioscope Catalyst (Bruker) was used to analyze the surface topography of photodynamically treated and untreated bacterial strains. Cells were imaged with a scan rate of 0.5 Hz. The scan size was 3 µm. We used a ScanAssyst-FLUID + silicon tip on a nitride lever with a resonant frequency of 100–200 kHz and a spring constant of 0.7 N m^−1^. AFM surface images were acquired in noncontact mode. For topographic images of both treated and untreated cells, the mean diameter was measured using the imaging software Gwydion 2.40. For each sample (control as well as treated samples), an average of 50 cells was imaged to ascertain the effect of photodynamic treatment on cell surface morphology. Analysis was carried out in triplicate samples for 3.24 J/cm^2^. This light dose was selected based on the result of antibacterial testing.

### Statistical analysis

The presented data were expressed as the mean and standard deviation (SD). Differences between two independent groups (the light and the dark) were determined by the independent two-sample t-test. Values with *p* values less than 0.05 were considered statistically significant. All statistical analyses were conducted using IBM SPSS Statistics for Windows, Version 23.0. Armonk, NY: IBM Corp. The level of significance of the test is marked denoted by asterisks: *p < 0.05 (significant), **p < 0.01 (very significant), ***p < 0.001 (extremely significant).

## Results

3.1. Photodynamic inactivation of methicillin-resistant *S. aureus*, ESBL-producing *K. pneumoniae*, *P. aeruginosa* and *E. faecalis* at room temperature.

**Figure 1:**
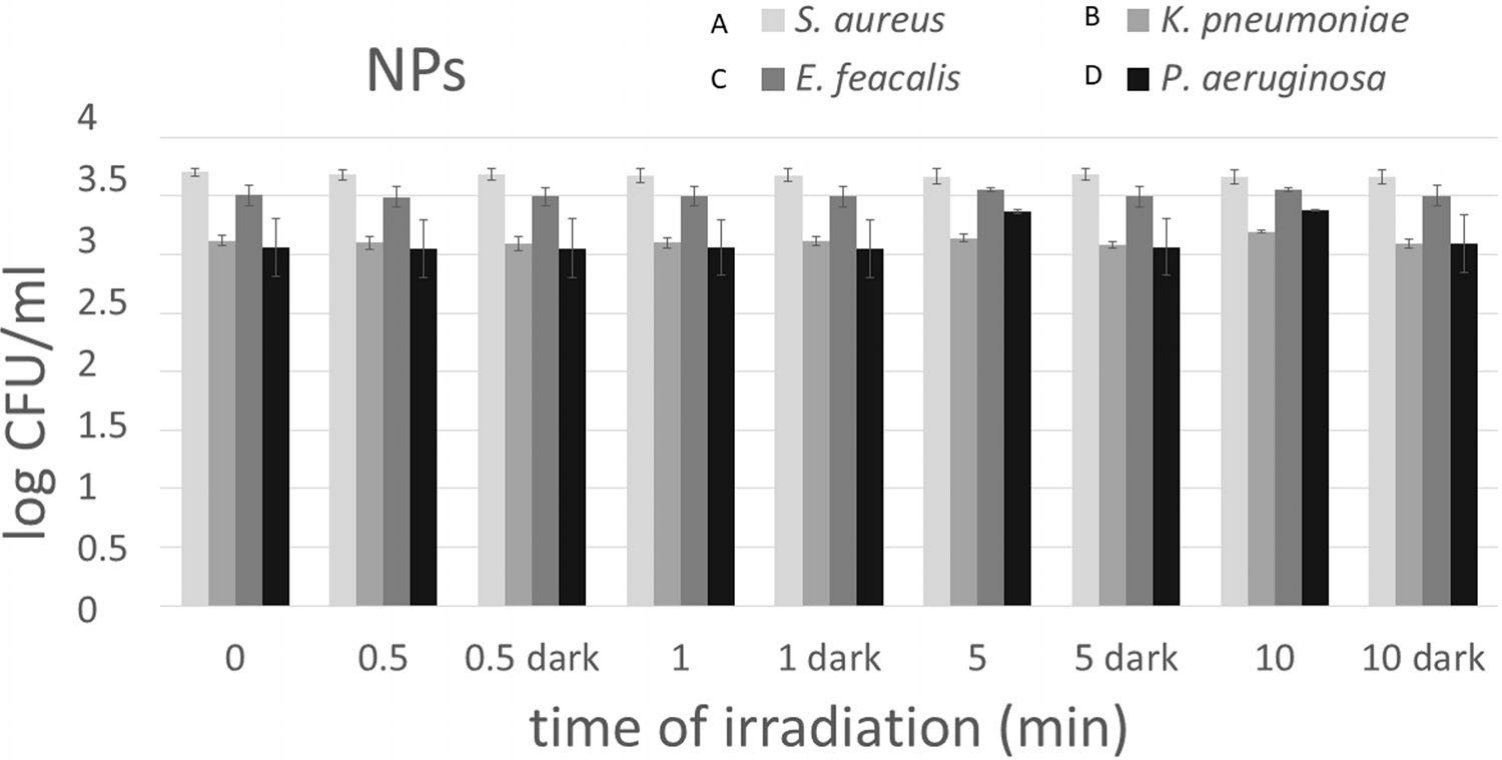
Photoinduced antibacterial activity of the polystyrene NPs (1.5 × 10^12^ NPs/ml) at different dose of irradiation. NPs dispersions (3 × 10^12^ NPs/ml) were mixed 1:1 with a dispersion of MRSA-*S. aureus* (**A**), ESBL-*K. pneumoniae* (**B**), *E. faecalis* (**C**) and *P. aeruginosa* (**D**). The results show the estimated average logarithm of the colony forming units per ml (log CFU/ml) observed on the agar plates for the irradiated and non-irradiated samples from 3 independent tests at room temperature.

**Figure 2:**
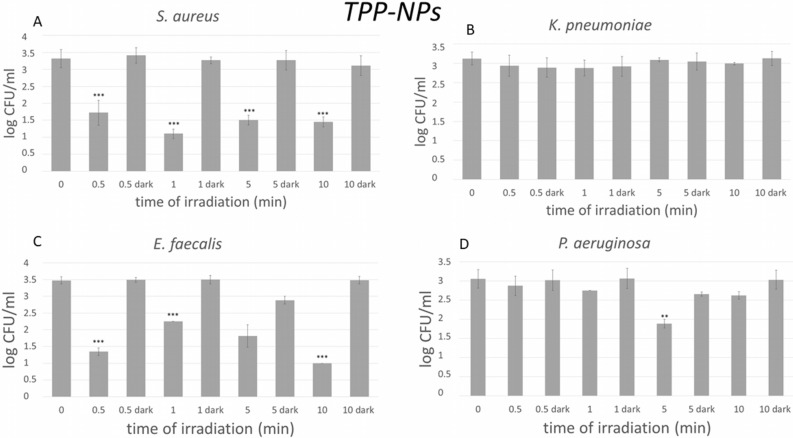
Photoinduced antibacterial activity of the TPP-NPs (1.5 × 10^12^ NPs/ml) at different light dose. TPP-NPs dispersions (3 × 10^12^ NPs/ml) were mixed 1:1 with a dispersion of MRSA-*S. aureus* (**A**), ESBL-*K. pneumoniae* (**B**), *E. faecalis* (**C**) and *P. aeruginosa* (**D**). Samples of bacteria with TPP-NPs were exposed to the irradiation for 0.5, 1, 5 and 10 min, which correspond to the specific doses of irradiation of 1.62, 3.24, 16.2 and 32.4 J/cm^2^, respectively. The results show the estimated average logarithm of the colony forming units (CFUs) observed on the agar plates for the irradiated and nonirradiated samples from 3 independent tests at room temperature. The level of significance of the test is often denoted by asterisks: *p < 0.05 (significant), **p < 0.01 (very significant), ***p < 0.001 (extremely significant).

The cytotoxicity of NPs without TPP and TPP-NPs with different light doses on methicillin-resistant *S. aureus,* ESBL-producing *K. pneumoniae, P. aeruginosa* and *E. faecalis* was evaluated by measuring the colony forming units (CFUs). The photoinactivation results (Figs. 1, 2) show the estimated average of log CFU/ml observed on the agar plates with the irradiated and nonirradiated samples from 3 independent experiments at room temperature.

No antibacterial effect was found using NPs without TPP or using TPP-NPs without irradiation toward any of the bacterial strains used. In the case of TPP-NPs, the antibacterial effect increased with increasing light doses.

Under light exposure, while a significant reduction of Gram-positive bacterial strains were inactivated, there was almost no antibacterial effect on ESBL-producing *K. pneumoniae* (Gram-negative bacterial strain) (Fig. 2B). The previous study confirms that ESBL-producing *K. pneumoniae* is highly resistant bacterial strains^34,35^.

We can conclude that Gram-positive bacterial strains are much more sensitive to PDI with TPP-NPs than Gram-negative bacteria under the same conditions.

PDI is highly efficient for MRSA. After very short light irradiation, 0.5 min (1.62 J/cm^2^), the number of colonies decreased by approximately 2 log CFU/ml compared to the dark control. Similar results were observed for ENTF. In contrast, there was no significant reduction in Gram-negative bacterial strains. For PSEA, the reduction in CFU/ml was observed only at 5 min exposure time. Moreover, there was no significant reduction in ESBL using 0.5 min irradiation.

### AFM of TPP-NPs

**Figure 3:**
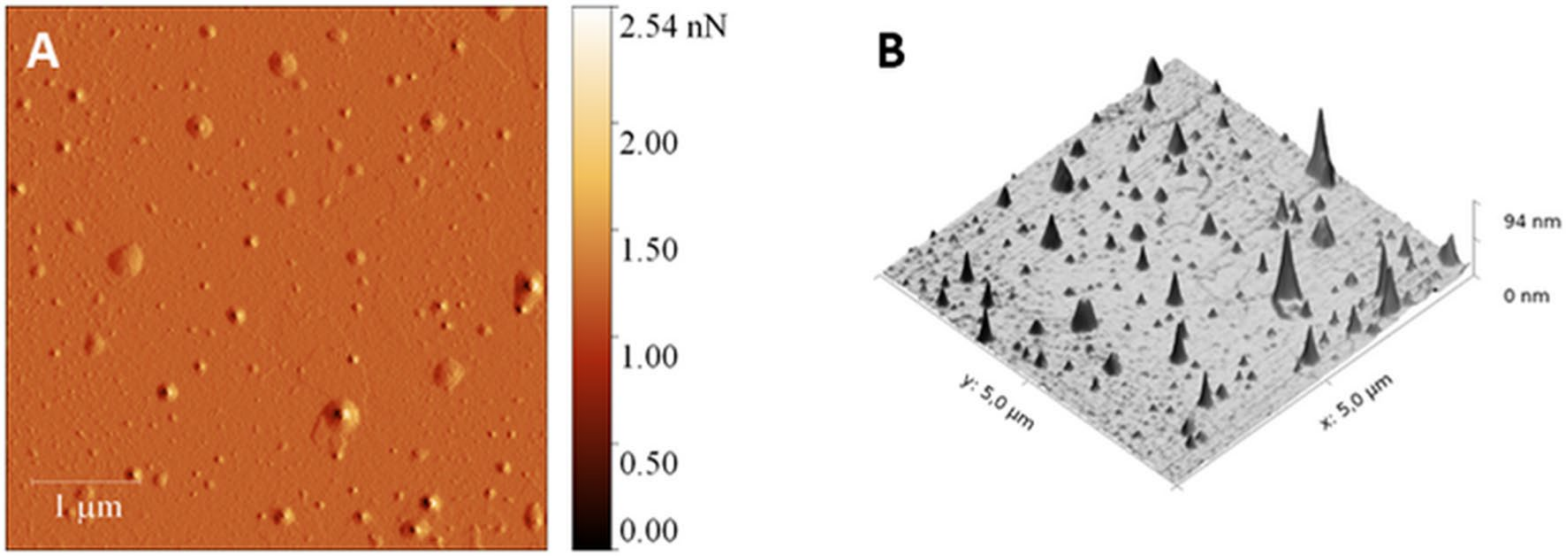
AFM 2D topography (**A**) and the corresponding 3D reconstructions (**B**) images of TPP-NPs. Scan area: 3.3 µm × 3.3 µm). Images were processed by Gwydion 2.40.

The surface morphology of TPP-NPs was recorded using AFM. The two- and three-dimensional topography of the TPP-NPs is shown in Fig. 3. Scans were acquired in noncontact mode, and the scan size was 3.3 µm. The scan rate was 0.5 Hz. AFM images were processed by Gwydion 2.40. Typically spherical shape of TPP-NPs with a broad distribution was observed. According DLS the average size of TPP-NPs was 15 ± 7 nm in diameter.

### AFM of photodynamically treated Gram-positive bacterial strains (MRSA and ENTF)

**Figure 4:**
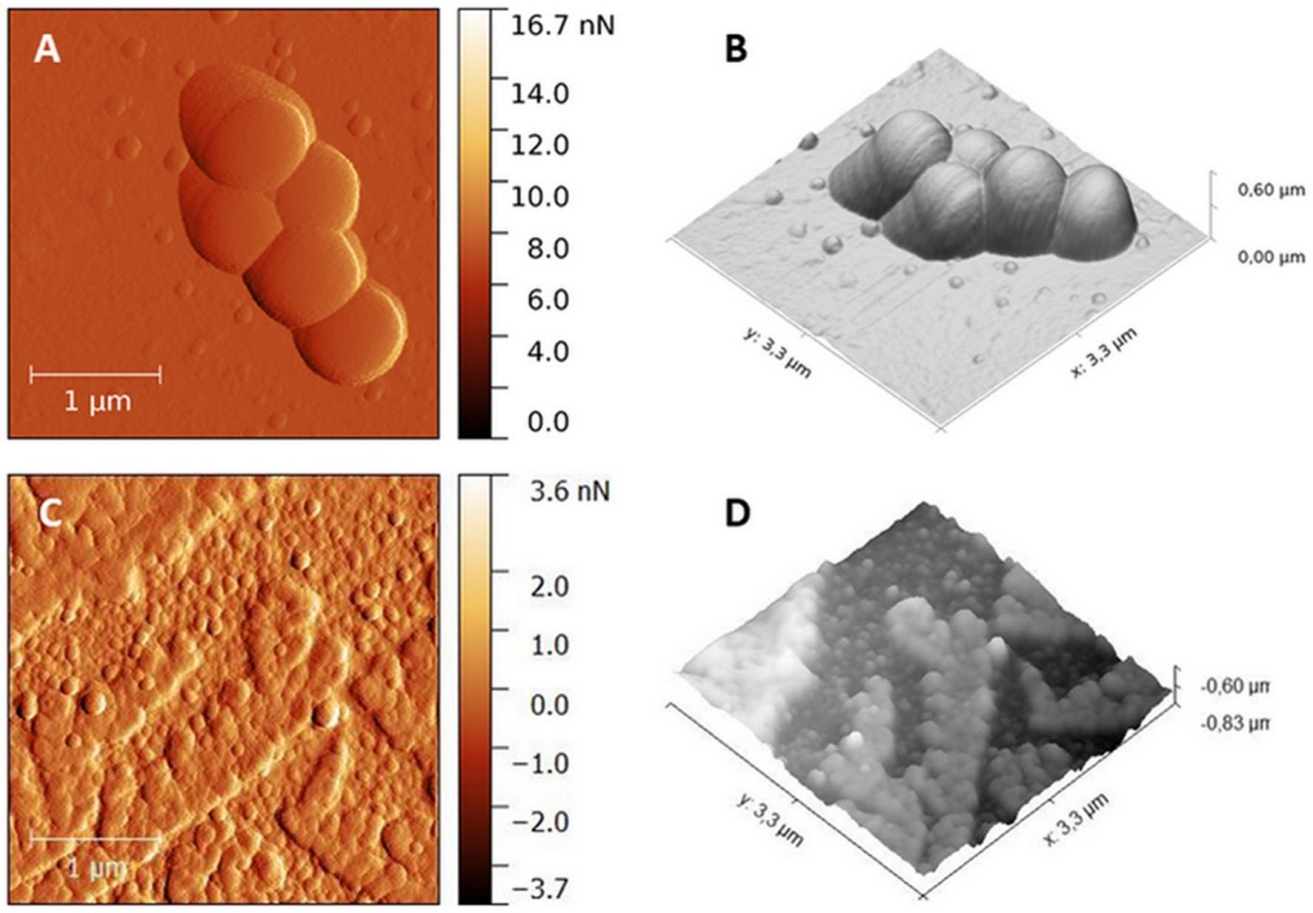
AFM 2D topography (left panel) and the corresponding 3D reconstructions (right panel) images of Methicillin-resistant *S. aureus* cells before therapy (**A**,**B**, scan area: 3.3 µm × 3.3 µm) and after treatment by TPP-NPs with irradiated light dose of 3.24 J/cm2 (**C**,**D**, scan area: 3.3 µm × 3.3 µm). Images were processed by Gwydion 2.40.

The AFM images of methicillin-resistant *S. aureus* treated and untreated with the TPP-NPs before and after irradiation, with certain light doses causing death of bacterial strains (1 min irradiation corresponds to 3.24 J/cm^2^), are shown in Fig. 4, (2D and 3D reconstructed). Images of the untreated cells with the TPP-NPs revealed cocci in clusters, which is a typical morphology of methicillin-resistant *S. aureus* (Fig. 4A,B).

The median height profile measured by AFM before and after PDI was 384.05 nm and 193.86 nm, respectively. In contrast, the morphology of the irradiated cells displays significant differences (Fig. 4C,D). The surface of the cells irradiated with 3.24 J/cm^2^ was rough. The bacterial cells were smaller, their membranes are disrupted and the cell contents are spilled.

**Figure 5:**
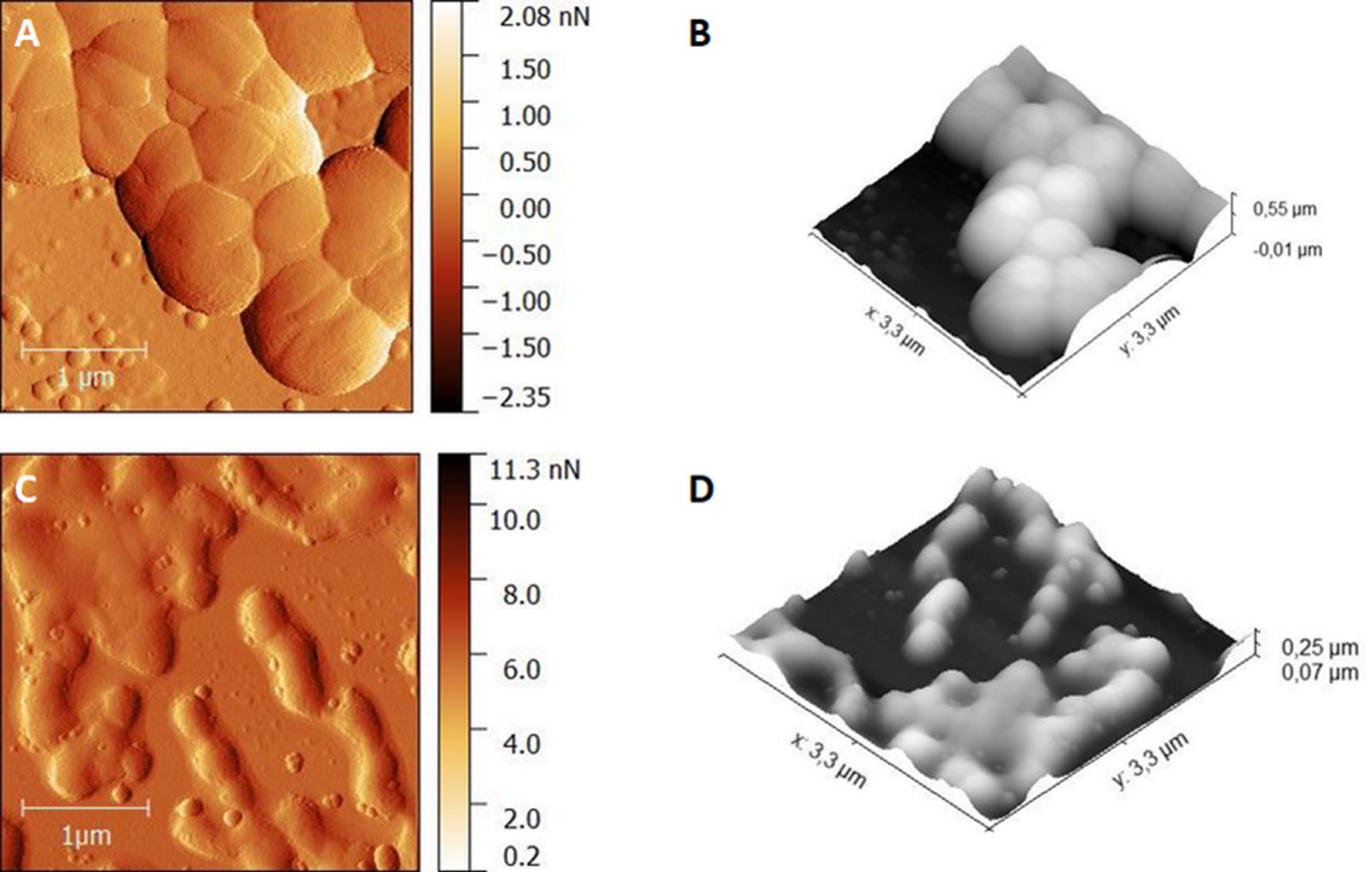
AFM 2D topografy (left panel) ant the corresponding 3D reconstructions (right panel) images of* E. feacalis* cells before therapy (**A**,**B**, scan area: 3.3 µm × 3.3 µm) and after treatment by TPP-NPs with irradiated light dose of 3.24 J/cm^2^ (**C**,**D**, scan area: 3.3 µm × 3.3 µm). Images were processed by Gwydion 2.40.

*E. faecalis* is a Gram-positive coccus bacterium that often grows in pairs (diplococci) or short chains (Fig. 5A,B). The median height profile before and after PDI was 216.5 nm and 208.48 nm, respectively. The small size cells with disturbed membranes and leaked contents of *E. faecalis* were obtained after PDI treatment using a light dose of 3.24 J/cm^2^ (Fig. 5C,D).

### AFM of photodynamically treated Gram-negative bacterial strains (ESBL and PSEA)

**Figure 6:**
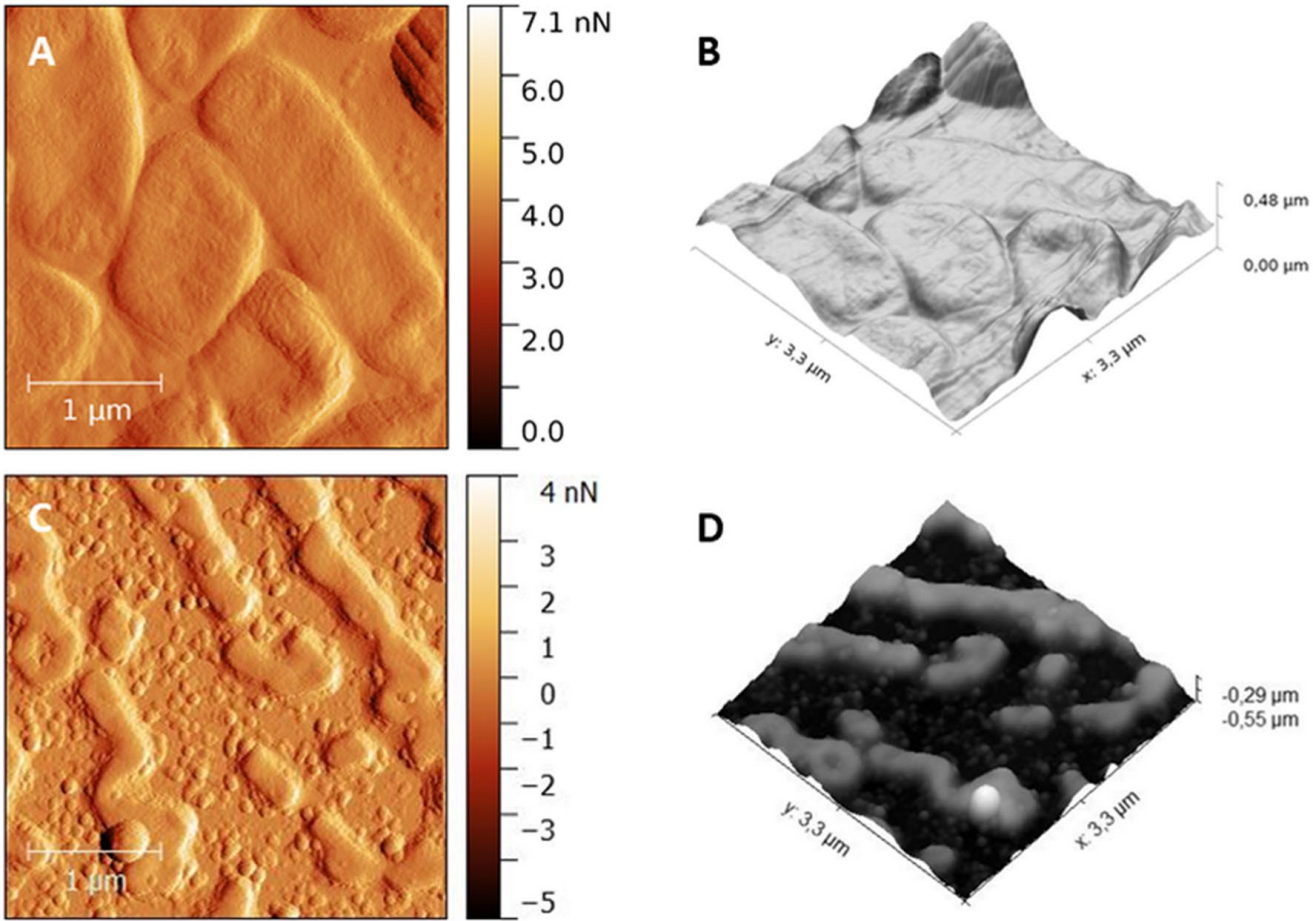
AFM 2D topografy (left panel) ant the corresponding 3D reconstructions (right panel) images of ESBL-producing *K. pneumoniae* cells before therapy (**A**,**B**, scan area: 3.3 µm × 3.3 µm) and after treatment by TPP-NPs with irradiated light dose of 3.24 J/cm^2^ (**C**,**D**, scan area: 3.3 µm × 3.3 µm). Images were processed by Gwydion 2.40.

The two- and three-dimensional reconstructed AFM image of ESBL-producing *K. pneumoniae* treated with the TPP-NPs before and after light exposure are shown. Healthy cells have an oval-like shape with regular and smooth surfaces (Fig. 6A,B). The median height profile before and after PDI was 226.85 nm and 136.65 nm, respectively. The cells damaged by the TPP-NPs mediated by PDI were found to have irregular and bleb-like protrusions on their surface (Fig. 6C,D).

**Figure 7:**
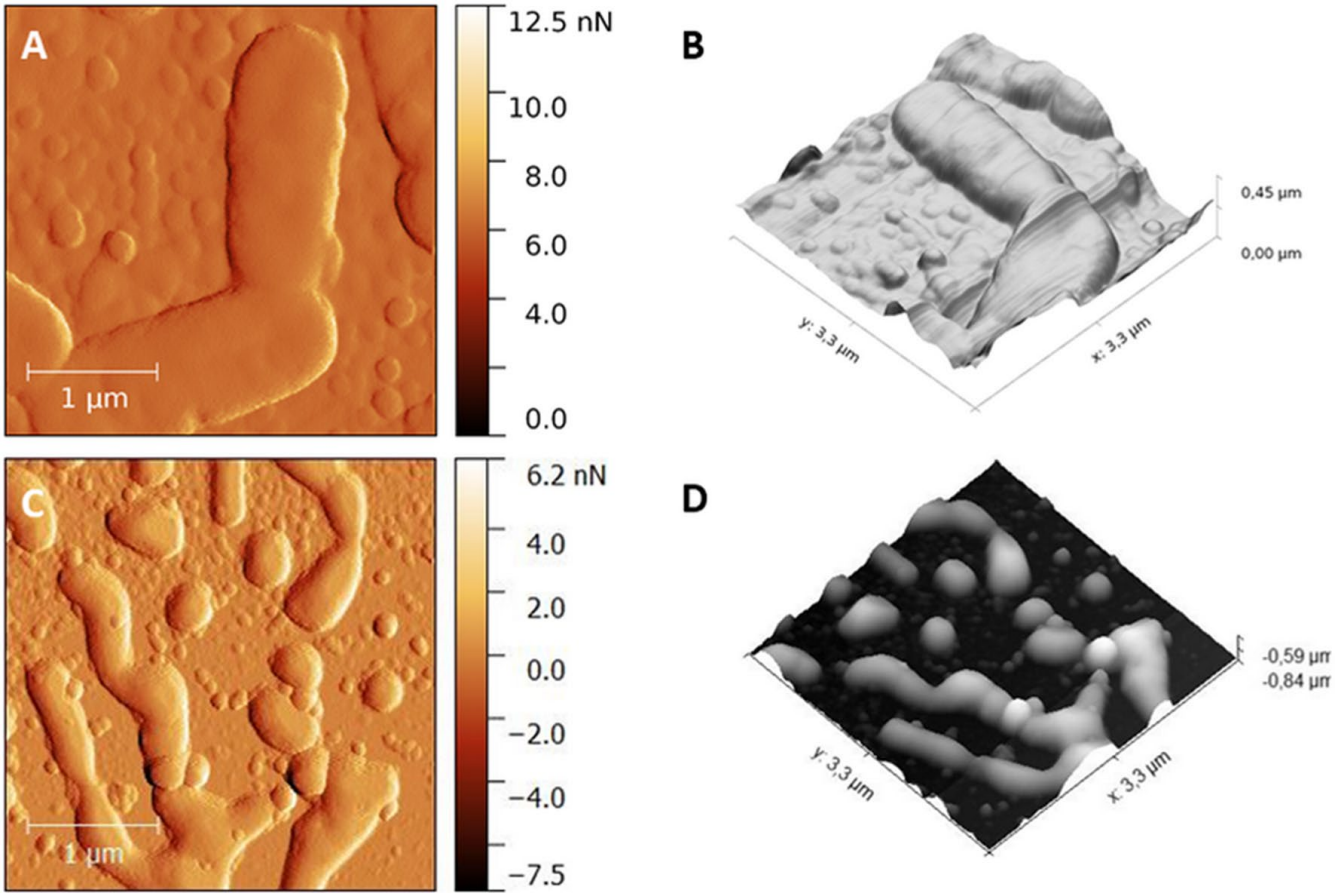
AFM 2D topografy (left panel) ant the corresponding 3D reconstructions (right panel) images of *P.aeruginosa* cells before therapy (**A**,**B**, scan area: 3.3 µm × 3.3 µm) and after treatment by TPP-NPs with irradiated light dose of 3.24 J/cm^2^ (**C**,**D**, scan area: 3.3 µm × 3.3 µm). Images were processed by Gwydion 2.40.

*P. aeruginosa* is a rod-shaped Gram-negative bacterium (Fig. 7A,B) with a slime layer that can cause diseases in plants and animals, including humans. Shrunken bacteria were observed after treatment with the TPP-NPs activated by visible light. (Fig. 7C,D). The median height profile before and after PDI was 335.1 nm and 169.69 nm, respectively.

## Discussion

Generally, the efficacy of PDI on bacteria depends on type of photosensitizer and strain of bacteria. Free water soluble photosensitizers need to meet special criteria to enter the bacteria if not abetted by permeability enhancers such as EDTA. As example, small photosensitizers like methylene blue as well as positively charged ones are promising for PDI, especially in combination with permeability enhancers. But, it has to be note, that every antibacterial mechanism relying on the presence of molecules inside bacterial cells is connected with a risk to induce the development of further resistance^36^. Recently it was proved that photosensitizers can exhibit strong phototoxic effect even without entering the cells and therefore eluding this risk. The high phototoxicity without entry of the photosensitizer into the cells can only be explained by damage to cell walls from outside^37^. It is generally believed that photosensitizers bounded on/in supporting matrix are often acting this way^38,39^. This inspired us to developed stable dispersion of sulfonated polystyrene nanoparticles with encapsulated photosensitizer (TPP-NPs). The TPP-NPs with monomeric TPP with high quantum yield of O_2_(^1^Δ_g_) have a negatively charged surface due to extensive sulfonation, which prevents aggregation in aqueous environments and allows to travel and release of O_2_(^1^Δ_g_) in close proximity to the chemical/biological targets. The polystyrene core is transparent to visible light and has a high oxygen diffusion coefficient. The sulfonated character not only ensures the stability of NPs, but also mimics anionic photosensitizers with limiting abilities to enter into the bacterial cells often possessing negative charges^31^.

In this study we used relatively small NPs (15 ± 7 nm). In general, smaller photoactive NPs photogenerating O_2_(^1^Δ_g_) or other ROS exhibit higher PDI^40^, due to the higher ratio “surface-to –volume” ratio compared to larger NPs. As a result, more efficient photogeneration of O_2_(^1^Δ_g_) or other ROS can be observed, which in turn inactivates essential biomolecules such as DNA, proteins and lipids of bacteria^41^. The detail antibacterial mechanism is complex, but comprise the disintegration of bacteria cell walls. One of the possibilities of disrupting cell walls is the external photooxidation of mainly wall lipids by ROS from NPs. Moreover, ROS generation has been shown to also act against the cell built-in antioxidant cellular wall defense mechanisms^42^. Another mechanism includes the direct interaction of NPs with a bacterial cell which can lead to membrane damage by NPs, which is sometimes followed by their penetration into the cell. Some studies show that adsorption on the cell wall followed by its disintegration is the basic mechanism of their toxicity^40^. Binding of NP to the cell wall leads to its depolarization and the wall becomes more permeable. Thus, the cell wall is destroyed first, followed by penetration of NPs. Subsequently, ROS are formed, inhibiting ATP production and DNA replication.

One of the main reason of this study was to find if it is possible to use PDI of NPs generating O_2_(^1^Δ_g_) for Gram-negative and Gram-positive (including multi-resistant) bacteria under the present mild (non-extreme) conditions (in respect of the time and the dose of irradiation and relatively low concentration of bacteria). NPs photogenerating highly cytotoxic O_2_(^1^Δ_g_) have some benefits but also limitation. At high concentration of bacteria, near-lying bacteria shield/protect the other bacteria from oxidation/cytotoxic effect of O_2_(^1^Δ_g_) with very short lifetime and diffusion pathway. So, with increasing bacterial concentration the PDI will decrease. In this study we used relatively low concentration of bacteria to see photoinactivation without this “shielding” effect.

Our results of dose-dependent experiments presented in Fig. 2 show a higher inactivation effect on Gram-positive bacteria than Gram-negative bacteria at the concentration of 1.5 × 10^12^ NPs/ml. The results are in accordance with a previous study showing that Gram-positive bacteria are more sensitive to PDI^39^. We observed that the effective inhibitory dose of irradiation at the concentration of 1.5 × 10^12^ NPs/ml on methicillin-resistant *S. aureus* and *E. faecalis* was 1.62 J/cm^2^, while on *P. aeruginosa*, the effective inhibitory dose of irradiation was ten times higher, 16.2 J/cm^2^.

The cell wall of Gram-positive bacteria is quite simple. It contains a high amount of peptidogycan and teichoic acids. Additionally, bacteria have no outer cell membrane. The Gram–negative bacteria have more complex cell wall. The wall is comprised of an outer and an inner membrane separated by a periplasmic space with thin peptidoglycan layer. The outer cell membrane is made by two lipid bilayers containing phospholipids, carbohydrates and proteins^43,44^. Due to these differences, a higher dose of irradiation generating more O_2_(^1^Δ_g_) is required to inactivate Gram-negative bacteria compared with Gram-positive bacteria^45^. Our results shows that TPP-NPs can damage only sensitive Gram-negative bacterial strains (PSEA). For ESBL producing *K. pneumonia* we did not observed any antibacterial effect. *K. pneumoniae*, unlike other strains tested, is encapsulated. The capsule consists mainly of polysaccharides and proteins, is located outside the cell wall and protects the cells from toxic substances.

Many authors^46–48^ reported that Gram-positive bacteria can be readily photoinactivated by O_2_(^1^Δ_g_), on the other hand, cell wall of Gram-negative species acts as an effective barrier that prevents the photooxidation and/or penetration of many photosensitive dyes. Using our negatively charged singlet oxygen-generating NPs, we found similar results. MRSA is very sensitive to the TPP-NPs mediated PDI, but ESBL-producing *K. pneumoniae* survived even with a high dose of irradiation (10 min, 32.4 J/cm^2^).

Marked differences in the topographies of Gram-negative and Gram-positive bacteria were detected in this study by AFM. Before and after PDI we analyzed 50 cells of each bacterial strain and we measured their height profile by AFM at the light dose of 3.24 J/cm^2^. After PDI, the height profiles of the bacteria were reduced in all tested bacterial strains. The Mann–Whitney U-test showed that pre-treatment values for all bacteria were statistically significantly higher than after the treatment, *p* < 0.0001 for all bacteria (Fig. S2).

The morphological changes between Gram-positive and Gram-negative bacterial strains were different. For Gram-positive bacterial strains, similar morphological changes were observed. All tested cells exhibited reduction of size. Their surfaces were wrinkled with disturbed membranes and leaked contents of the bacteria.

For all Gram-negative bacteria we found shrunken effect. Cells exhibited irregular and bleb-like protrusions on their surfaces. Different degree of morphological damage was found for *P.aeruginosa*, some cells had a disturbed membrane and leaked their contents. For ESBL-producing *K. pneumoniae*, reduced cells were observed compared to the cells before PDI, but no membrane damage and no leaked contents. This is probably due to the fact that ESBL-producing *K. pneumoniae* is an encapsulated bacterial strain and PDI mediated by TPP-NPs is not effective.

The topographic alterations induced by PDI were observed for light dose of 3.24 J/cm^2^, causing equivalent phototoxicity in both types of bacteria except *K. pneumoniae*. The AFM results showed that the PDI with TPP-NPs induced changes in the cell surface of bacteria. An increased roughness and a disruption of the cell surface indicate damage and disorganization of cell walls, which may be a consequence of a destabilization of the peptidoglycan network and/or oxidative damage induced in the membrane components^49,50^.

The AFM morphological data suggested that the main O_2_(^1^Δ_g_) target induced by TPP-NP irradiation is the cell envelope. AFM can be utilized as a powerful, sensitive tool to assess the efficacy of antibacterial agents and explore the drug delivery mechanism^26^. The AFM was used as a sensitive and rapid visual tool for studying the interactions between bacteria and singlet oxygen-generating NPs.

## Conclusion

In summary, we report the AFM characterization and antibacterial properties of sulfonated polystyrene nanoparticles with encapsulated hydrophobic TPP (5,10,15,20-tetraphenylporphyrin) photosensitizers (TPP-NPs). We found that singlet oxygen-generating TPP-NPs are promising photosensitizing agents for PDI in several antibacterial applications triggered by visible light. In our in vitro study, two Gram-positive and two Gram-negative bacterial strains were used. Methicillin-resistant *S. aureus* (MRSA) and *Enterococcus faecalis* CCM 4224 (ENTF) were the Gram-positive bacterial strains used, and ESBL-producing *Klebsiella pneumoniae* (ESBL) and *Pseudomonas aeruginosa* CCM 3955 (PSEA) were the Gram-negative bacterial strains used. The Gram-positive bacterial strains were found to be highly sensitive and easily inactivated by TPP-NPs. AFM can be used as a sensitive tool to evaluate the efficacy of these photodynamic antibacterial agents.

## Supplementary information

Supplementary information.

## References

[1] Farkas J, Doyle M, Beuchat L (2007). Physical Methods of Food Preservation. Food Microbiology: Fundamentals and Frontiers.

[2] Dobrynin D, Fridman G, Friedman G, Fridman A (2009). Physical and biological mechanisms of direct plasma interaction with living tissue. New J. Phys..

[3] Dillow AK, Dehghani F, Hrkach JS, Foster NR, Langer R (1999). Bacterial inactivation by using near- and supercritical carbon dioxide. PNAS.

[4] Ye M, Sun M, Huang D, Zhang Z, Zhang H, Zhang S (2019). A review of bacteriophage therapy for pathogenic bacteria inactivation inthe soil environment. Environ. Int..

[5] Mohanty S, Mishra S, Jena P, Jacob B, Sarkar B, Sonawane A (2012). An investigation on the antibacterial, cytotoxic and antibiofilm efficacy of starch-stabilized silver nanoparticles. Nanomed. Nanotechnol..

[6] Salomoni, R., Léo, P., Rodrigues, M. Antibacterial activity of silver nanoparticles (AgNPs) in *Staphylococcus aureus* and cytotoxicity effect in mammalian cells. the battle against microbial pathogens: Basic science, technological advances and educational programs. 851–857, (Formatex 2015).

[7] Benov L (2015). Photodynamic therapy: Current status and future directions. Med. Princ. Pract..

[8] Dai T, Huang YY, Hamblin MR (2009). Photodynamic therapy for localized infections—state of the art. Photodiagn. Photodyn..

[9] Wainwright M (1998). Photodynamic antimicrobial chemotherapy (PACT). J. Antimicrob Chemoth..

[10] Hamblin M (2016). Antimicrobial photodynamic inactivation: A bright new technique to kill resistant microbes. Curr. Opin. Microbiol..

[11] George S, Hamblin MR, Kishen A (2009). Uptake pathways of anionic and cationic hotosensitizers into bacteria. Photochem Photobiol Sci..

[12] Fotinos N, Convert M, Piffaretti JC, Gurny R, Lange N (2008). Effects on gram-negative and gram-positive bacteria mediated by 5-aminolevulinic acid and 5-aminolevulinic acid derivatives. Antimicrob Agents Chemother..

[13] Ghorbani J, Rahban D, Aghamiri S, Teymouri A, Bahador A (2018). Photosensitizers in antibacterial photodynamic therapy: An overview. Laser Ther..

[14] Amos-Tautua BM, Songca SP, Oluwafemi OS (2019). Application of porphyrins in antibacterial photodynamic therapy. Molecules.

[15] Procházková K, Zelinger Z, Lang K, Kubát P (2004). Meso-tetratolylporphyrins substituted by pyridinium groups: Aggregation, photophysical properties and complexation with DNA. J. Phys. Org. Chem..

[16] Zhang L, Gu FX, Chan JM, Wang AZ, Langer RS, Farokhzad OC (2008). Nanoparticles in medicine: Therapeutic applications and developments. Clin. Pharmacol. Ther..

[17] Kubát P, Henke P, Berzédiová V, Štěpánek M, Lang K, Mosinger J (2017). Nanoparticles with embedded porphyrin photosensitizers for photooxidation reactions and continuous oxygen sensing. ACS Appl. Mater. Interfaces..

[18] Kubát P, Henke P, Raya RK, Štěpánek M, Mosinger J (2020). Polystyrene and poly(ethylene glycol)-b-poly(ε-caprolactone) nanoparticles with porphyrins: Structure, size, and photooxidation properties. Langmuir.

[19] Usacheva MN, Teichert MC, Biel MA (2001). Comparison of the methylene blue and toluidine blue photobactericidal efficacy against Gram positive and Gram negative microorganisms. Laser Surg Med..

[20] Usacheva MN, Teichert MC, Biel MA (2003). The role of the methylene blue and toluidine blue monomers and dimers in the photoinactivation of bacteria. J. Photoch. Photobiol B..

[21] Schär-Zammaretti P, Ubbink J (2003). The cell wall of lactic acid bacteria: Surface constituents and macromolecular conformations. Biophys. J..

[22] Dörr T, Moynihan PJ, Mayer C (2019). Editorial: Bacterial cell wall structure and dynamics. Front. Microbiol..

[23] Vollmer W, Blanot D, De Pedro MA (2008). Peptidoglycan structure and architecture. FEMS Microbiol. Rev..

[24] Salmon-Divon M, Nitzan Y, Malik Z (2004). Mechanistic aspects of *Escherichia coli* photodynamic inactivation by cationic tetra-meso (N-methylpyridyl) porphine. Photochem. Photobiol. Sci..

[25] Ubbink J, Schär-Zammaretti P (2005). Probing bacterial interactions: Integrated approaches combining atomic force microscopy electron microscopy and biophysical techniques. Micron.

[26] Sahu K, Bansal H, Mukherjee Ch, Sharma M, Gupta PK (2009). Atomic force microscopic study on morphological alterations induced by photodynamic action of Toluidine Blue O in *Staphylococcus aureus* and *Escherichia coli*. J. Photoch. Photobiol. B..

[27] da Silva A, Teschke O (2005). Dynamics of the antimicrobial peptide PGLa action on *Escherichia coli* monitored by atomic force microscopy. World J. Microbiol. Biotechnol..

[28] Bolshakova AV, Kiselyova OI, Filonov AS, Frolova OY, Lyubchenko YL, Yaminsky IV (2001). Comparative study of bacteria with an atomic force microscopy operating in different modes. Ultramicroscopy.

[29] Sullivan CJ, Morrell JL, Allisona DP, Doktycz MJ (2005). Mounting of *Escherichia coli* spheroplasts for AFM imaging. Ultramicroscopy.

[30] Dolanský J, Henke P, Malá Z, Žárská L, Kubát P, Mosinger J (2018). Antibacterial nitric oxide- and singlet oxygen-releasing polystyrene nanoparticles responsive to light and temperature triggers. Nanoscale.

[31] Henke P, Kirakci K, Kubát P, Fraiberk M, Forstová J, Mosinger J (2016). Antibacterial, antiviral and oxygen-sensing nanoparticles prepared from electrospun materials. ACS Appl. Mater. Interfaces..

[32] Tomecka, M., Bajgar, R., Kolarova, H.: Light source of uniform energy density to induce photodynamic phenomena in vitro cells. Czech patent CZ 302829 B6, (2011). https://patents.google.com/patent/CZ302829B6

[33] Robichon D, Girard JC, Cenatiempo Y, Cavellier JF (1999). Atomic force microscopy of dried or living bacteria. C. R. Acad. Sci. Ser. III Sci. Vie.

[34] Lee ChH, Su LH, Tang YF, Liu JW (2006). Treatment of ESBL-producing Klebsiella pneumoniae bacteraemia with carbapenems or flomoxef: A retrospective study and laboratory analysis of the isolates. J. Antimicrob. Chemoth..

[35] Navon-Venezia S, Kondratyeva K, Carattoli A (2017). Klebsiella pneumoniae: A major worldwide source and shuttle for antibiotic resistance. FEMS Microbiol. Rev..

[36] Preuß A, Zeugner L, Hackbarth S, Faustino M, Neves M, Cavaleiro J, Roeder B (2013). Photoinactivation of *Escherichia coli* (SURE2) without intracellular uptake of the photosensitizer. J. Appl. Microbiol..

[37] Nie X, Wu S, Mensah A, Lu K, Wei Q (2020). Carbon quantum dots embedded electrospun nanofibers for efficient antibacterial photodynamic inactivation. Mater. Sci. Eng. C..

[38] Henke P, Kozak H, Artemenko A, Kubát P, Forstová J, Mosinger J (2014). Superhydrophilic polystyrene nanofiber materials generating O2(1Δg): Postprocessing surface modifications toward efficient antibacterial effect. ACS Appl. Mater. Interfaces..

[39] Jin H, Huag X, Chen Y, Zhao H, Ye H, Huang F, Xing X, Cai J (2010). Photoinactivation effects of hematoporphyrin monomethyl ether on Gram-positive and -negative bacteria detected by atomic force microscopy. Appl. Microbial. Cell Physiol..

[40] Slavin YN, Asnis J, Häfeli UO, Bach H (2017). Metal nanoparticles: Understanding the mechanisms behind antibacterial activity. J. Nanobiotechnol..

[41] Karakoti AS, Hench LL, Seal S (2006). The potential toxicity of nanomaterials—the role of surfaces. JOM.

[42] Ramalingam B, Parandhaman T, Das SK (2016). Antibacterial effects of biosynthesized silver nanoparticles on surface ultrastructure and nanomechanical properties of Gram-negative bacteria viz. *Escherichia coli* and *Pseudomonas aeruginosa*. ACS Appl. Mater. Interfaces..

[43] Mai-Prochnow A, Clauson M, Hong J, Murphy AB (2016). Gram positive and Gram negative bacteria differ in their sensitivity to cold plasma. Sci. Rep..

[44] Baron, S. Medical microbiology. Galveston, Tex: University of Texas Medical Branch at Galveston (1996). 21413252

[45] Hamblin MR, Hasan T (2004). Photodynamic therapy: A new antimicrobial approach to infectious disease. Photochem. Photobiol. Science..

[46] Dahl TA, Midden WR, Hartman PE (1989). Comparison of killing of Gram-negative and Gram-positive bacteria by pure singlet oxygent. J. Bacteriol..

[47] Hamblin MR, O’Donnell DA, Murthy N, Rajagopalan K, Michaud N, Sherwood ME, Hasan T (2002). Polycationic photosensitizer conjugates: Effects of chain length and Gram classification on the photodynamic inactivation of bacteria. J. Antimicrob. Chemother..

[48] Ban S, Caruso E, Bucca L (2006). Antibacterial activity of tetraarylporphyrin photosensitizers: An in vitro study on Gram negative and Gram positive bacteria. J. Photochem. Photobiol. B.

[49] Katsui N, Tsuchido T, Hiramatsu R, Fujikawa S, Takano M, Shibasaki I (1982). Heatinduced blebbing vesiculation of the outer membrane of *Escherichia coli*. J. Bacteriol..

[50] Pillet F, Formosa-Dague C, Baaziz H, Dague E, Rols MP (2016). Cell wall as a target for bacteria inactivation by pulsed electric fields. Sci. Rep..

