# Supplementary material for: Photodynamic effect of TPP encapsulated in polystyrene nanoparticles toward multi-resistant pathogenic bacterial strains: AFM evaluation
Source: Sci Rep. 2021 Mar 24;11:6786. doi: 10.1038/s41598-021-85828-9 (PMC7990921; doi:10.1038/s41598-021-85828-9)
Supplement: Supplementary file 1 — Supplementary information. [file 41598_2021_85828_MOESM1_ESM.docx]

**Supplementary data**

**Photodynamic effect of TPP encapsulated in polystyrene nanoparticles toward multiresistant pathogenic bacterial strains: AFM evaluation**

Zuzana Malá ^a^, Ludmila Žárská ^a^, Lukáš Malina ^a^, Kateřina Langová ^a^, Renata Večeřová ^b^, Milan Kolář ^b^, Petr Henke ^c^, Jiří Mosinger ^c^, Hana Kolářová ^a^

^a^ *Department of Medical Biophysics, Faculty of Medicine and Dentistry, Palacky University in Olomouc, Czech Republic*

*^b^ Department of Microbiology, Faculty of Medicine and Dentistry, Palacky University in Olomouc, Czech Republic*

*^c^ Department of Inorganic Chemistry, Faculty of Science, Charles University, Czech Republic*


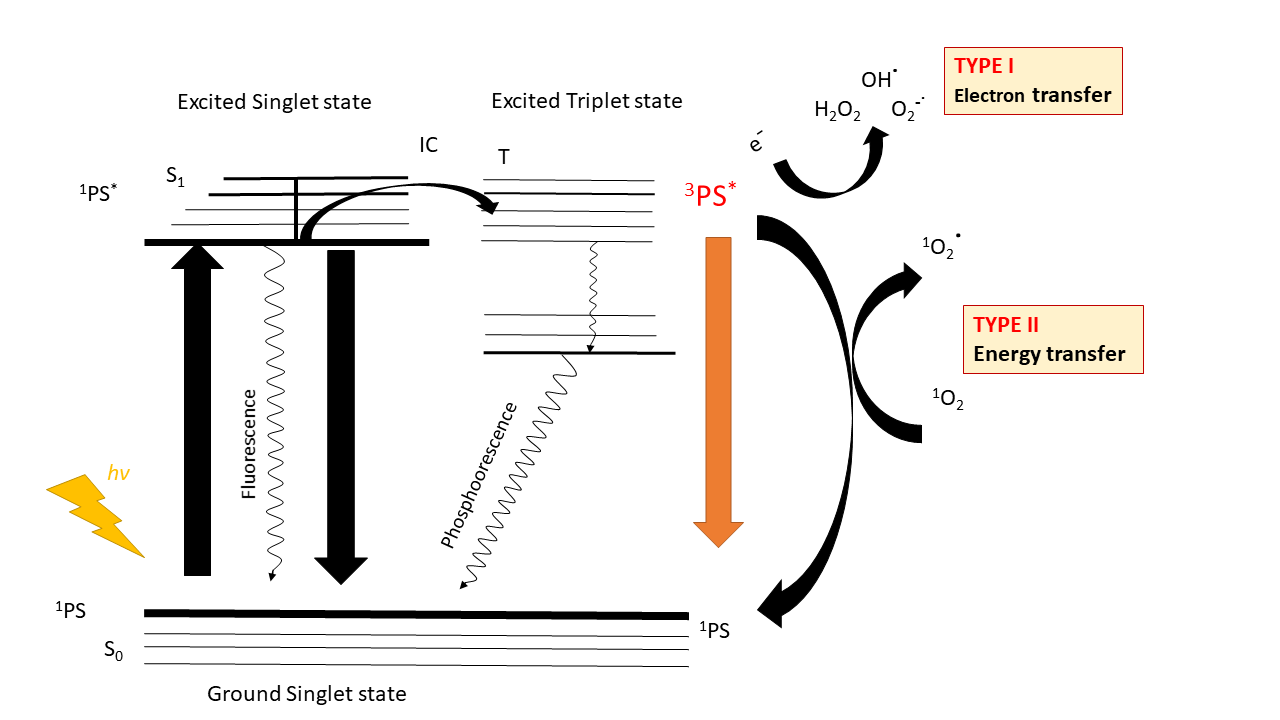


Fig. S1. Schematic mechanism of photodynamic reaction type 1 (electron transfer) and type 2 (energy transfer). Schematic Jablonski's diagram showing the mechanism of action of PDT. Following light absorption, the PS reaches an excited singlet state. After intersystem crossing, the PS, now in a triplet excited state, can react in two different ways. First, the triplet PS can undergo an electron transfer reaction between some surrounding electron donor or electron acceptor molecule, to form radical anion or a radical cation. These radicals can further react with oxygen to form superoxide (O2-˙), hydrogen peroxide (H2O2) and hydroxyl radicals (HO˙). Second, PS in its triplet state can react directly with oxygen through energy transfer, generating singlet oxygen.


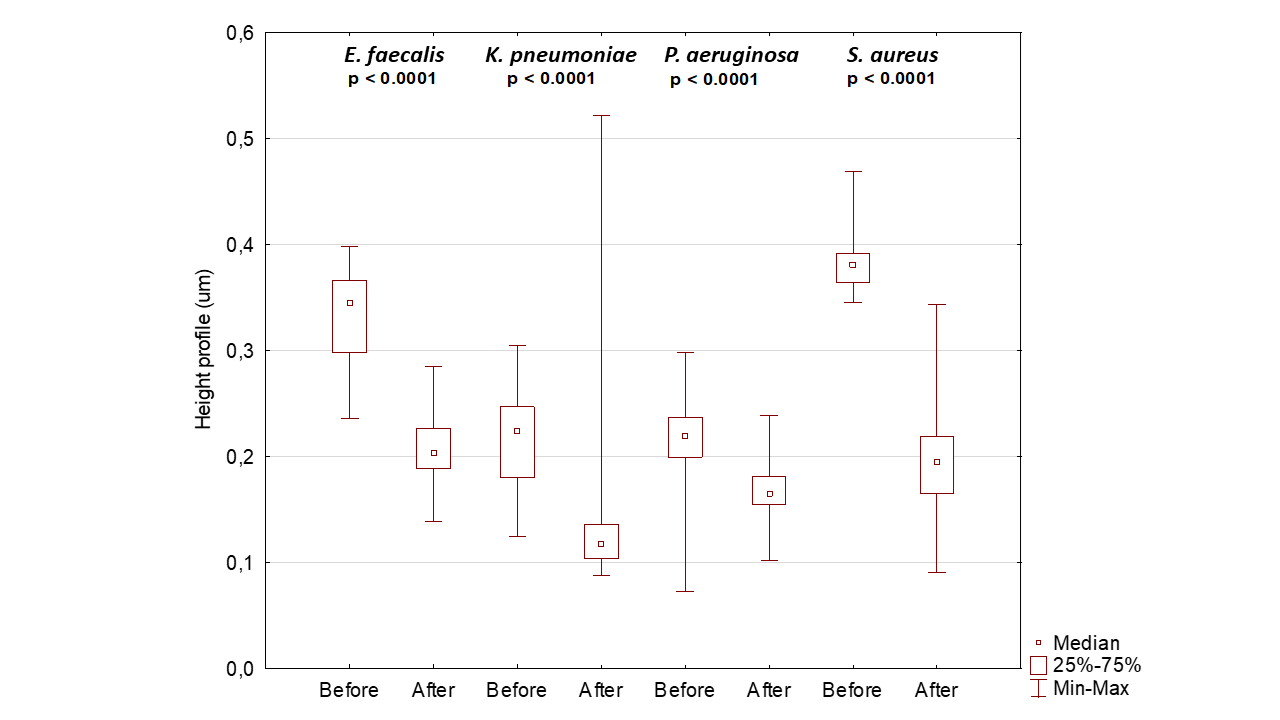


Fig. S2.The statistical anylysis of height profile of all tested bacterial strains. „Before“ represented untreated bacteria and „after“ represented bacteria exposed TPP-NPs with 3.24 J/cm2. The Mann-Whitney U-test showed that pre-treatment values ​​for all bacteria were statistically significantly higher than after treatment, p <0.0001 for all bacteria.
